# Anorectal Malformations: Ideal Surgery Timing to Reduce Incontinence and Optimize QoL

**DOI:** 10.3390/children10020404

**Published:** 2023-02-18

**Authors:** Gloria Pelizzo, Carlotta Paola Maria Canonica, Francesca Destro, Milena Meroni, Dario Rizzo, Lorena Canazza, Giorgio Giuseppe Orlando Selvaggio, Eleonora Durante, Gianvincenzo Zuccotti, Valeria Calcaterra

**Affiliations:** 1 Department of Biomedical and Clinical Science, University of Milan, 20157 Milan, Italy; 2 Pediatric Surgery Department, Buzzi Children’s Hospital, 20154 Milan, Italy; 3 Pediatric Department, “Vittore Buzzi” Children’s Hospital, 20154 Milan, Italy; 4 Pediatrics and Adolescentology Unit, Department of Internal Medicine, University of Pavia, 27100 Pavia, Italy

**Keywords:** anorectal malformation, quality of life, children, incontinence

## Abstract

Anorectal malformations (ARMs) are rare and involve a wide spectrum of malformations. Prenatal diagnosis is often incomplete, and the diagnostic pathway is started during the newborn period to identify the type of malformation and the correct treatment. This retrospective study included patients between 8 and 18 y.o. diagnosed with ARM, referring to Our Clinic. We proposed two questionnaires, Rintala Bowel Function Score and the Fecal Incontinence Quality of Life Scale, and we defined four groups referring to surgical timing (age in months < 3, 3–6, 6–9, >9). In total, 74 patients were recruited (mean age 13.05 ± 2.80 y.o.), and data analysis showed a significant relationship between comorbidity and surgical timing. Moreover, timing was related to outcome in terms of fecal continence (better if surgery performed before 3 months) and Quality of Life (QoL). QoL, however, is influenced by other factors (emotional and social life, psychological sphere and take of care of chronic disease). We considered rehabilitation programs, more often practiced by children who underwent surgery after 9 months, to maintain an appropriate relational life. This study highlights the importance of surgical timing as the first step of a multidisciplinary follow-up, taking care of the child in every phase of his growth, tailored to the single patient.

## 1. Introduction

Anorectal malformations (ARMs) are a rare disease, occurring in 1:4000/5000 neonates, that involves the last tract of the gastrointestinal tube. The defect origin is from embryogenic development, from the hindgut to the definitive rectum and anus and relative structures, such as perineal body and urogenital organs. These types of malformations are a wide spectrum of disease, from the simpler defects to more complex such as cloaca [1]. Prenatal diagnosis is very difficult, and often relates to higher defect with undirect signs detectable during pregnancy; definitive diagnosis is obtained at birth, with perineal inspection [2,3]. From early life, these children embark on a diagnostic pathway in order to identify the type of malformation and the correct treatment, along with the comorbidities associated. During recent years, surgeons have been more focused on long term follow-up, including both fecal continence and Quality of Life (QoL) [4,5,6].

ARMs involve a high rate of morbidity during infanthood and adolescence; this brings psychological disorders, such as discriminations that reflect in the emotional sphere [7]. Incontinence is a social issue, involving children’s auto-determination about their own perception of QoL (embarrassment, self-confidence, problems at school) [8]. Moreover, it is important to consider other factors affecting QoL in these patients: frequent hospitalizations, repeated surgeries and, during the postoperative follow-up program, the child and the family have to deal with the dilatation program [9]. Moreover, ARMs could be associated with other malformations, involving the need for other procedures affecting QoL [10,11,12,13]. For these reasons, it is fundamental to give incontinence and QoL a crucial role from the diagnosis of ARMs throughout long-term follow-up.

The aim of the study was to identify the ideal surgery timing to obtain better continence and QoL. Although it is established that certain types of malformations relate to a better prognosis in terms of continence, this is not always true about QoL [14]. Surgery timing could be an important factor to consider at the time of diagnosis to achieve the best result for the child’s health and well-being.

## 2. Patients and Methods

### 2.1. Patients

We conducted a retrospective single center study at our Paediatric Surgery Department (Buzzi Children’s Hospital, Milan, Italy), between September 2020 and May 2021.

Patient data (demographics, pre and perinatal data, comorbidities, type of ARMs, surgical management, postoperative complications, and follow-up) were collected by medical records, hospital servers and operating room reports; missing data were obtained by parent interview.

### 2.2. Methods

The type of ARM was classified on the basis of the Krickenbeck International Classification [15]. Patients were divided into four groups based on age at the time of definitive repair—under the age of 3 months, between 3 and 6 months, between 6 and 9 months and older than 9 months of age.

Surgical interventions were collected and classified according to the type of procedure as follows: posterior sagittal anorectal pull-through (PSARP), laparoscopic-assisted anorectal pull-through (LAARP) and cutback.

Two questionnaires (see section below) were submitted to families and/or patients contacted through an online form. Maneuverers to control bowel movements, dietary modifications, the use of laxatives and bowel management were recorded at follow-up and investigated with an optional section of the questionnaire.

Two questionnaires were chosen for this study. They are both reported and validated. In particular, the second one has a validated translation in Italian, which made it more suitable for us. The Rintala Bowel Function Score [16] and an Italian validated translation of the Fecal Incontinence Quality of Life Scale (FIQL) [17] were used as questionnaires. For patients <12 years of age, the questionnaires were modified in order to exclude sexual-life queries. The Rintala Bowel Function Score is composed by seven questions, each one with a possible score from 0 to 3 (except for the frequency of defecation, that goes from 0 to 2). We considered a total score of 18 or more as an indicator of normal bowel continence. The FIQL is composed of 29 questions, divided into four domains: Lifestyle (10 questions), Coping/Behaviour (9 questions), Depression/Self Perception (7 questions), Embarrassment (3 questions). Maximum global score, which is the sum of single scores, is 119.

#### 2.2.1. Rintala Bowel Function Score

This questionnaire has the characteristic of being simple and well understandable by the patients. It could be administrated by the clinician or filled in by the patient himself. It is a brief sequence of scored answers about fecal incontinence, consistence of the stool and “accidents”, which gives the physician a clear image of the bowel situation and of the required intervention.

#### 2.2.2. FIQL—Fecal Incontinence Quality of Life Scale

The FIQL was chosen because it focuses on more complex aspects related to daily life and permits to establish a particular level of personal satisfaction derived from the Quality of Life. It investigates a more private sphere of each patient, often involving caregivers. For this reason, we left a sort of autonomy in filling the forms, meaning that patients needed to express their opinion but parents and/or guardians could help them when required.

### 2.3. Statistical Analysis

The statistical analysis was performed using IBM SPSS^®^ statistics 28.0. Data were described with the mean, median, standard deviation (SD), standard error, upper and lower bound of confidence interval at 95%, maximum and minimum of every group. For every test, a *p*-value < 0.05 was considered statistically significant. An inferential statistic was applied through analysis of the variance (ANOVA) between groups. Levine and Welch tests were used to test the feasibility.

## 3. Results

### 3.1. Features of the Patients

A total of 133/172 patients managed for ARMs between 8 and 18 years were offered to join the study and have been contacted; 92/133 accepted to join the study. Among them, 74 (39 males, 35 females, mean age 13.05 ± 2.80 years) met the inclusion criteria and were considered for the analysis.

According to Krickenbeck International Classification: 37/74 (50.0%) patients had rectoperineal fistula (RP), 11/74 (14.9%) had rectovestibular fistula (RV), 9/74 (12.2%) bulbar RU and 6/74 (8.1%) prostatic RU, 6/74 (8.1%) had cloaca (C), 2/74 (2.7%) had rectoneckbladder fistula (RNB), 1/74 (1.3%) had imperforate anus without fistula and 2/74 (2.7%) had rectal stenosis/atresia.

Associated comorbidities were identified in 33 patients (33/74, 44.6%), of those 10/74 (13.5%) have a specific syndrome (VACTERL, T21, Currarino).

Detailed features of associated comorbidities are listed in Table 1.

### 3.2. Surgical Details

At birth, 31/74 patients (41.9%) had a colostomy (11 RU fistula both bulbar and prostatic, 6 C, 5 RV fistula, 4 RP fistula, 2 RNB fistula, 1 imperforate anus, 2 rectal stenosis) and 43/74 (58.1%) underwent definitive repair. We performed 67 PSARPs (67/74, 90.5%), 5 cutbacks (5/74, 6.8%), 1 LAARP (1/74, 1.3%).

We observed surgical complication in 5/74 patients (6.7%) out of our series (3 anal stenosis and 3 prolapses). Surgical complications were observed belatedly, though it was not relevant in the choice of the surgical timing.

Patients were divided into four groups based on the age at the time of definitive repair: <3 months of age (19/74, 25.7%), between three and six months of age (19/74, 25.7%), between six and nine months of age (15/74, 20.3%) and >9 months of age (21/74, 28.4%), as listed in Table 2.

In our sample, sex and type of malformation distribution showed similar features to those exhibited in the literature [1,18,19]. Patients treated before 3 months of age were mainly RP and RV fistulas; only five of them presented comorbidities. Among the group of patients who underwent surgery between 3 and 6 months of age, notably three patients with cloaca were found, and 73.7% of them had comorbidities. The group operated between 6 and 9 months was the smaller in number. The remaining 21 patients underwent surgery after 9 months of age; among them we could account for one imperforate anus without fistula and one rectal stenosis/atresia. Moreover, about half of them presented comorbidities.

In the last two groups, comorbidities (see Table 1) were identified in 4 (4/15, 26.7%) and 10 (10/21, 47.6%) patients, respectively.

### 3.3. Follow-Up and Questionnaire Results

Some of the patients, or their caregivers case of young children, filled in the form also with their daily life maneuvers to treat constipation and incontinence. The need for dietary modifications, laxatives and bowel management in the four groups is reported in Table 3. None obtained statistical significance.

### 3.4. Questionnaire Results

The results of the Rintala Bowel Function Score and FIQL are reported in Table 4 and Table 5. The association between age at the time of surgery and Rintala score presented a chi2 *p*-value = 0.067 and an ANOVA *p*-value = 0.151, very close to the cut-off of statistical significance, probably due to the small number of the sample. Total FIQL displayed statistical significance (*p* = 0.011) for association with age at time of surgery, with higher scores reported in the group of 3–6 months. Lower scores are reported for groups <3 months and >9 months. Single domain scores do not show statistically significant differences and their mean value is similar in the four groups.

## 4. Discussion

ARMs include a wide spectrum of diseases affecting the anus, the rectum and the urogenital tract. Affected patients may be indistinguishable from normal subjects or present with severe functional alterations. Under the classification point of view, many systems have been proposed during the years; nowadays, a straightforward and descriptive classification, indicating anatomically where the fistula and the rectum end, is preferred. This type of classification, proposed in 2005 (Krickenbeck, Germany), has prognostic and therapeutic value. The most common malformations are rectourethral bulbar fistula in males and vestibular fistula in females. In both sexes, the second most common type is the perineal fistula. The cloaca represents the most complex ARM, in which the rectum, urethra and vagina end in the same common channel (short or long, the former with better prognosis). Clinical assessment at birth permits the identification of lower defects, but the anatomical precise definition requires additional radiological investigations, considering that neonatal physiology is peculiar, and it may require 18–24 h for the anatomy to be fully defined [1]. Another important aspect is related to the presence of associated anomalies, affecting up to 60–70% of ARM patients (30% of all patients have cardiac defects, >50% have urological abnormalities, 5–10% have esophageal/duodenal atresia) [1]. These associated anomalies deeply affect the prognosis and could complicate surgery. The precise definition of the anatomy and associated defects is fundamental to achieve the best outcomes. For all these reasons, despite progressive improvement in the clinical care of patients, there is the risk of short and long-term morbidity for both physical and psychological domains. Surgical possibilities include the primary definitive repair or the delayed reconstruction by performing a colostomy at birth. Multiple factors should be considered before deciding the management, including the type of malformation and associated anomalies, the clinical conditions of the patient, the experience of the surgeon and the team of health professionals taking care of the baby. Sometimes colostomy is performed as an urgent operation with the aim of reducing abdominal distention and preserving genital and urinary tracts. In selected patients, some surgeons prefer to delay the repair avoiding the colostomy, when the fistula opening permits a satisfactory fecal output. Less than 10% of patients will require an additional abdominal approach by either laparotomy or laparoscopy [1].

The primary end point of surgical and rehabilitation programs is the achievement of a socially acceptable fecal continence. The most challenging cases are those with severely altered anatomy and impaired function/physiology of the pelvic structure. Together with the type of malformation, it has been shown that there are several risk factors associated with fecal and urinary incontinence, such as sacral underdevelopment, cord anomalies, altered nerve supply and associated anomalies.

Recently, the advantages of an early anorectoplasty were pointed out, including the possibility to avoid colostomy, to reduce the number of operations and to obtain a better bowel function [20]. The feasibility and safety of the one-stage correction of high-variety ARMs, with acceptable or even fewer early postoperative complications, were also reported. The positive effects of neonatal surgery are likely to be related to the early relief of intestinal obstruction, the removal of the urinary tract connection with reduced risk of urological contamination and the early acquisition of a physiological defecation mechanism [21,22,23]. Peña, after describing the PSARP technique, indicated the first two months of life as the ideal timing for definitive correction [1].

After surgery, all ARM patients require long-term follow-up, starting from the first post-operative weeks. Early childhood maybe characterized by constipation or fecal incontinence, generally responding well to medical management. Delicate periods of growth and hormonal changes are to be treated with particular attention (potty training, school period and adolescence). Some patients have various degrees of muscular hypoplasia and require a strict protocol of take in charge of their condition. Bowel habits are usually a chronic condition that might improve, even though persisting throughout the whole life of the patient. Many authors underline the important role of dysmotility in constipation onset. Aggressive management is mandatory to prevent fecal impaction, intestinal dilatation and soiling resulting from pseudo-incontinence [16]. Some teams include the role of the rehabilitation therapist for the management of patients with encouraging results in terms of improving sphincter and anorectal function [13]. There are various types of rehabilitation programs, including functional exercises and mechanical stimulation (i.e., sacral and tibial nerve stimulation, physio-kinesis therapy, superficial electrostimulation, biofeedback) in association with behavioral adjustments. These approaches are not always recommended (the presence of sacral malformation or anatomical anomalies could impair the efficacy of the treatment, as well as patient’s compliance) and should be evaluated on a single base. The concept of a tailored approach should address the choice of individual management. Considering the need of a long term take in charge, a new clinical field, related to transitional care, has been improving. The transitional care involves different medical and surgical health care professionals, including gynecologists, andrologists, general surgeons of the adult and psychologists, in order to assist these patients into their adult life. Another important topic relates to the awareness of the child and of the adolescent towards their medical condition, often unknown.

Hartman et al. [8] pointed out the importance of auto-determination in affecting the self-perception of QoL. Most patients succeed in having a good social integration, but there are also reports about discrimination during childhood [7].

Other studies revealed how the presence of comorbidities have an impact on QoL, mostly because of repeated hospitalizations and psychophysical discomfort for the growing child [24,25]. It is clear how the complexity of ARMs influences fecal and urinary continence and psychosocial spheres at different stages of growth. Psychosocial issues may show up in the early stages of life and increase during growth when untreated [26]. In particular, these problems can become exacerbated in the transitional age and adolescence. These phases of life are particularly critical for the development of the sexual sphere, the emotional and cognitive evolution, the presence of physical changes and the pursuit of a new role into family and society.

Therefore, QoL evaluation should be part of the global assessment of patients during follow-up, as suggested by numerous studies [13,27,28,29].

In the current study, we chose a sample with an age range from 8 to 18 years, taking into account both the pre-adolescence period and the transitional age. A recent literature review [26] of articles published between 1980 and 2019 exhibits many struggles with peer and self-acceptance during transition to school and adolescence together with reported feelings of embarrassment and shame, traumatic memories related to medical care. Behavioral concerns have been found in 18–27% of ARM children. Adolescents with ARMs seem to have a double risk of emotional problems compared to patients with other chronic diseases. Psychological problems are common: suicidal thoughts have been reported in 15% of cases, maladjustment in 18%, depression/anxiety in 27% and aggressive behaviors in 27%. Strong medical support and familial response positively affect the emotional and psychosocial development during growth [6,25,26,30].

In our series, QoL perception was globally satisfactory: the FIQL average related to total answers given was 99.5 (83.6% of the maximum score of 119). This positive result may be favored by immediate and constant patient care.

Surgery timing affects different parameters, and we observed differences between different age groups at time of surgery.

Type of malformation is a statistically significant variable in determining the different age groups at time of surgery; in fact, more complex malformations often require colostomy at birth, postdating surgery time [18,31]. Moreover, colostomy at birth reported a significant result regarding age groups at time of surgery.

Comorbidities affect surgery timing significantly, with surgery most frequently being observed between 3 and 6 months among patients with comorbidities. Distribution of the type of malformation throughout the groups reveals that the presence of comorbidity is associated with surgery timing regardless of the type of malformation. Moreover, the presence of comorbidity appeared to be relevant for surgery timing.

Our center provides follow-up care from the immediate post-natal period through adolescence and young adult age; this includes screening for correlated conditions and the possibility to treat them in the earlier stages of life, reducing patient morbidity and mortality. However, this process brings a high number of outpatient examinations and a high number of health care professionals involved, while it has an important impact over self-perception.

No significant difference in terms of diet has been noticed among the different groups during our analysis; this factor is very susceptible to family habits, so it may not be solid.

Laxatives are used more in the group that underwent surgery between 3 and 6 months.

More interestingly, bowel management maneuverers are mostly used in the group of children who had surgery after 9 months and least in the group who had surgery before 3 months. There is no statistical evidence, but it is a close-up result (probably due to small sample size); a possible explanation may be that children who had surgery later in life are more susceptible to need a bowel management program in order to meet their social and personal needs [32,33].

Two fundamental results were highlighted through questionnaires.

Fecal continence and QoL are strictly related to each other, considering different aspects such as the anatomical one (type of surgery) but also the sensory and motility.

The type of surgery was not analyzed because almost the entire sample had a PSARP surgery.

Sensory and motility were inspected by two different points of view through the submissions of two different forms.

Regarding fecal incontinence (Rintala Questionnaire), most scores above 18 (fecal continence) are reported in the group of patients who had surgery before 3 months. Further analysis of the group (<3 months) composition reveals how early surgery for less complex malformations, such as rectoperineal fistula (73.7%) and rectovestibular fistula (15.8%), could lead to a better outcome for fecal continence. In addition, observation of scores from the same malformation in other age groups reveals most scores below 17, implying a lower continence level.

The advantages of an early surgery were investigated by different points of view, and other studies bring results in favor of this choice [20,23,34].

From the analysis of FIQL questionnaire replies, Quality of Life scores are distributed differently among groups of age at time of surgery; these results are statistically significant. Most lower scores are represented in the group who had surgery within 3 months. At this point, Quality of Life seems to be influenced by a spectrum of variables, not only by fecal continence. Box plot analysis shows an inhomogeneity in scores, mostly in the groups below 3 months and above 9 months. Considering higher scores, obtained by groups who underwent surgery between 3–6 months and 6–9 months, these are composed mostly by complex malformations. In this scenario, more complex malformations treated between 3 and 9 months of age gained a better Quality of Life outcome.

About FIQL subdomains, the analysis was inconclusive, probably also related to the lack of specific replies in the questionnaire filling.

The retrospective nature of the study limits its accuracy, and many patients were lost at follow-up, gaining fragmentary information about actual state, which could have led to a bias in the selection process. Moreover, the study was conducted during the COVID-19 pandemic, not allowing to meet children in person and letting them fill the forms with families’ help, leading to possible interpretation bias and less autonomy in the answers. Finally, sample size was limited.

## 5. Conclusions

Our experience was meaningful from different points of view. First, there is a correlation between surgery timing and outcome in relation to fecal continence.

Indeed, fecal continence seems to be positively affected by surgery timing—within 3 months for non-complex malformations, within 12 months for more complex malformations.

Quality of Life, though, looks to be related to other life aspects, such as life habits, psychological stress, and chronic disease take of care; these seem to have an important role as determinants for the QoL [7,8].

This study reveals how surgery is the first step in a course of treatment, from birth to adult life, through puberty and adolescence. Results about fecal continence and Quality of Life show the need for a comprehensive and customized path for each patient, beginning with surgery time choice and pursuing the best follow-up care over all aspects.

## Figures and Tables

**Table 1:** Associated comorbidities and relative percentages on affected patients, classified according to age at time of surgery.

Comorbidity	Time of Surgery, *N* %				
	Total	<3 Months	3–6 Months	6–9 Months	>9 Months
Urologic	13 39.4%	1	6	3	3
Orthopedic/neurological/vertebral	12 36.3%	4	4	2	2
Gastroenterological/respiratory	9 27.3%	2	3	2	2
Cardiac	6 13.2%	0	3	0	3
Syndromes (VACTERL, T21, Currarino)	10 30.3%	2	4	2	2

**Table 2:** Features of the patients according to the time of definitive repair.

Recorded Features	Time of Definitive Surgery, *N* %				*p*-Value ANOVA	*p*-Value Chi Quadro
	<3 Months *N* = 19	3–6 Months *N* = 19	6–9 Months *N* = 15	>9 Months *N* = 21		
Sex						
Female	5 26.3%	10 52.6%	8 53.4%	12 57.1%		
Male	14 73.7%	9 47.3%	7 46.7%	9 42.8%		
Type of Malformation					*p* = 0.038	
Rectoperineal fistula	14 73.7%	5 26.3%	7 46.7%	11 52.4%		
Rectovestibular fistula	3 15.8%	3 15.8%	3 20.0%	2 9.5%		
Rectourethral fistula						
Bulbar	1 5.3%	3 15.8%	2 13.3%	3 14.3%		
Prostatic	1 5.3%	4 21.1%	-	1 4.8%		
Cloaca	-	3 15.8%	2 13.3%	1 4.8%		
Rectoneckbladder fistula	-	1 5.3%	-	1 4.8%		
Imperforate anus without fistula	-	-	-	1 4.8%		
Rectal stenosis/atresia	-	-	1 6.7%	1 4.8%		
Comorbidities						
Yes/No	5 26.3%	14 73.7%	4 26.7%	10 47.6%	*p* = 0.016	*p* = 0.011
Colostomy at birth Yes/No	2 10.5%	12 63.2%	7 46.7%	10 47.6%	*p* = 0.007	*p* = 0.009
Type of surgery					*p* = 0.297	*p* = 0.364
PSARP	17 89.5%	19 100.0%	11 73.4%	20 95.2%		
Cutback	2 10.5%	-	2 13.4%	1 4.8%		
Pull-Through	-	-	1 6.7%	-		
Surgical complications (prolapse, stenosis)	-	3 15.8%	1 6.7%	1 4.8%		

PSARP = posterior sagittal anorectal pull-through; LAARP = laparoscopic-assisted anorectal pull-through.

**Table 3:** Maneuverers used to treat constipation and fecal incontinence in the four groups.

	Diet *p* = 0.960	Laxatives *p* = 0.058	Bowel Management *p* = 0.132
Total, *N* %	16 21.6%	15 20.3%	23 31.1%
<3 months, *N* %	4	3	2
3–6 months, *N* %	4	7	6
6–9 months, *N* %	4	0	6
>9 months, *N* %	4	5	9

**Table 4:** Rintala results.

	SCORE < 17	SCORE > 18
<3 months *N* = 19	7 36.8%	12 63.2%
3–6 months *N* = 19	14 73.7%	5 26.3%
6–9 months *N* = 15	10 66.7%	5 33.3%
>9 months *N* = 21	15 71.4%	6 28.6%

**Table 5:** Fecal Incontinence Quality of Life Scale results.

	Mean/Median (Min–Max)				ANOVA *p*-Value
	<3 Months	3–6 Months	6–9 Months	>9 Months	
Total score	52.32/59.00 (7–107)	85.67/99.00 (7–112)	82.47/93.00 (34–115)	67.15/91.50 (7–108)	*p* = 0.011
Lifestyle	3.7583/4.0000 (2.40–4.00)	3.7505/3.9000 (2.63–4.00)	3.6590/3.8500 (2.89–4.00)	3.5511/3.8000 (2.00–4.00)	
Coping/ Behavior	3.5009/3.8000 (1.75–4.00)	3.3207/3.5278 (1.14–4.00)	3.4629/3.8000 (1.60–4.00)	3.5357/3.7500 (1.88–4.00)	
Depression/ Self-perception	3.7860/3.8333 (1.83–4.50)	3.6722/3.8333 (2.50–4.33)	3.5106/3.8333 (1.86–4.00)	3.7567/3.8333 (2.50–4.50)	
Embarrassment	3.3056/4.0000 (1.00–4.00)	3.3549/3.6667 (1.33–4.00)	3.2647/3.6667 (1.33–4.0)	3.3452/3.6667 (1.50–4.00)	

## Data Availability

The data presented in this study are available on request from the corresponding author.
